# Current scenario of endourological treatment of kidney stones in brazil: results of a national survey

**DOI:** 10.1590/S1677-5538.IBJU.2019.0363

**Published:** 2020-02-20

**Authors:** Rafael Haddad Astolfi, Raphael Carrera, Nelson Gattas, Ricardo Bertolla, Fabio Sepulveda, Ernesto Reggio, Alex Elton Meller

**Affiliations:** 1 Universidade Federal de São Paulo Disciplina de Urologia São PauloSP Brasil Disciplina de Urologia, Universidade Federal de São Paulo - UNIFESP, São Paulo, SP, Brasil; 2 Escola Paulista de Medicina - UNIFESP São PauloSP Brasil Escola Paulista de Medicina - UNIFESP, São Paulo, SP, Brasil; 3 Seção de Reprodução Humana Universidade Federal de São Paulo Departamento de Cirurgia São PauloSP Brasil Departamento de Cirurgia, Divisâo de Urologia, Seção de Reprodução Humana Universidade Federal de São Paulo - UNIFESP, São Paulo, SP, Brasil; 4 Universidade Estadual do Sudoeste da Bahia Disciplina de Urologia Vitória da ConquistaBA Brasil Disciplina de Urologia, Universidade Estadual do Sudoeste da Bahia - UESB, Vitória da Conquista, BA, Brasil; 5 Uroclínica de Joinville JoinvilleSC Brasil Uroclínica de Joinville, Joinville, SC, Brasil

**Keywords:** Kidney Calculi, Epidemiology, Ureteroscopy, Therapeutics

## Abstract

**Objective:**

To elucidate the current scenario of endourology in Brazil for the treatment of urinary lithiasis, with an emphasis on regional differences and the reasons why certain techniques are still underutilized.

**Materials and Methods:**

An electronic questionnaire was sent by email to the 4,745 members of the Brazilian Urological Society (BSU) in 2016 to collect information on the 3 main endourological procedures used in the treatment of nephrolithiasis: Semi-rigid ureteroscopy (URS), Flexible ureteroscopy (F-URS) and percutaneous nephrolithotripsy (PCNL).

**Results:**

A total of 1,267 urologists answered the questionnaire. It was observed that the vast majority perform URS (95.6%), while 80.2% perform F-URS and only 72.1% perform PCNL. Regarding the surgical volume, most perform up to 10 procedures per month (73.4% to 88.2%) and the main impediment was the lack of patients with the pathology (42.1% to 67.7%). The lack of equipment or hospital infrastructure was one of the main limiting factors for rigid (23%) and flexible (38.1%) URS, mainly in the North and Northeast regions of the country. Regarding PCNL, most of them reported lack of practical experience in the method (29.9%). Finally, most urologists expressed interest in taking courses in endourology.

**Conclusion:**

Ureteroscopy, rigid or flexible, is already well established in the country, requiring the direction of more resources for its practice, especially in less developed regions. Regarding PCNL a significant part of Brazilian urologists still lack practical experience in this procedure, emphasizing the need for greater investment in teaching this technique.

## INTRODUCTION

Nephrolithiasis is one of the most frequent urological diseases, with a significant burden on public health system and on patient's quality of life. Its global prevalence has increased over the years, from 2 to 20% depending on the geographical region, being higher in developed countries (1-3). In the USA, more than 8.8% of the population is affected by the disease (4), with an annual cost of up to 5.3 billion dollars, including direct costs of treatment and indirect secondary costs due to loss of work productivity (5), since kidney stones affect in particularly active economic people (6). In Brazil, according to DATASUS, 52 million reais (approximately US$ 13 million) have been spent in 2017 to treat kidney stones (7).

Over the last 30 years, treatment changed profoundly due to technological advances in minimally invasive techniques, that almost completely replaced open surgeris (1, 8, 9). The development of digital endoscopes and improvements of Holmium:Yag lasers and hydrophilic and flexible devices mande endourological procedures safer and more efficient, reducing surgical morbidity while improving clinical results (10, 11). In line with this world tendency, in the last 15 years, the number of endoscopic procedures increased greatly in Brazil, probably due to better training of young urologists during residency and to a higher availability of endoscopic equipment in different health centers (12).

The objective of this study was to clarify the current use of endourology on the treatment of kidney stones in Brazil, including regional differences and the reasons why some techniques are still underutilized. This information will aid the development of continuous education programs proposed by the Brazilian Urological Society (BSU), the improvement of endourology teaching in medical residency programs and better planning of resources distribution in different urological services in Brazil.

## MATERIAL AND METHODS

### Demographic data colection

Many sources were utilized to gather our data bank. After permission of the Brazilian Urological Society (BSU) to access its data, we have evaluated the total number of Brazilian members in debt or not, as well as the number of members of each Brazilian macro-region (North, Northeast, South, Southeast, Center-West). We have also evaluated the number of medical residency programs in Urology acredited by BSU in total and in each region. In order to estimate the number of urologists practicing in Brazil (members and non-members of BSU) we obtained data from the Federal Board of Medicine (13). The National Board of Medical Residencies (Conselho Nacional de Residencias Médicas - CNRM) provided the number of residency programs in urology, accredited or not by BSU, in total and in each Brazilian region. All information referred to 2018. In relation to general epidemiological characteristics, the databank of Instituto Brasileiro de Geografía e Estatística (IBGE - Brazilian Institute of Geography and Statistics) to determine the number of inhabitants in Brazil and the value of the Gross Internal Product (GPI) in total and according to the different regions. Finally, the HDI (Human Development Index) total and divided were obtaines from the databank of IPEA-Instituto de Pesquisa Economica Aplicada-Institute of Applied Economics Research-of 2016 (14).

### Research instrument

From January to September, 2016, a national survey was proposed by BSU to evaluate the current status of endourology in Brazil. An electronic questionnaire of 12 items (multiple choice) was sent by e-mail to all 4.745 current BSU members to collect demographic information and the practice of the three most used endourological procedures to treat kidney stones: semi-rigid ureteroscopy (URS), flexible ureteroscopy (F-URS), and percutaneous nephrolithotomy (PCNL). It was also asked to the respondents to estimate monthly number of each procedure and where he/she was located according to each of the five Brazilian regions (North, Northeast, South, Southeast and Center-West). Monthly rate of procedures was divided into three categories according to answer: <10 patients/month, 10-20 patients/month and >20 patients/month. If the respondents did not perform the procedure or considered the number insufficient, they were asked to point the reason why:1) Lack of equipment/hospital infrastructure; 2) Lack of theoretical knowledge of the method; 3) Lack of practical experience with the technique; 4) Lack of trained support staff; or 5) lack of patients with the disease. Finally, the respondents were asked to inform if they would participate in theoretical-practical courses ministered by experts in their regions, as well as which distance would they be willing to travel to participate (<50 km, 50-200 km, or >200 km). The answers were automatically and anonymously distributed in a database under confidentiality of the researcher (A.M.).

#### Statistical analysis

Statistical analysis was made by SPSS 17.0 software for Windows. Descriptive analysis was performed for continuous and categoric variables. When appropriate, frequencies were compared among groups using the Chi-Square test and, in that case, the maximum alpha error was set at 5%.

## RESULTS

### General data of urology in brazil

In 2018, 5.328 urologists were practicing in Brazil, 97.8% male and 2.2% female, with a median age of 48.7 years, representing 1.4% of the total of Brazilian medical doctors. Urologists were distributed unevenly in the Brazilian territory and were concentrated mainly in the Southeast region (52.2%) and in lesser number in the North region (4.3%). Among all Brazilian urologists, most were members of BSU (98.5%) and with lower frequency in the Center-West region (52.3%). In relation to the number of medical residency programs in urology (MRU), in 2018 there were 122 programs in Brazil, with a total of 142 positions per year, most located in the Southeast (57.4%) and with the lowest presence in the North region (4.1%). Among all, 80 MRU were accredited by BSU (65.6%) and in South and Center-West it was observed a higher proportion of accreditation according to total number (80% and 70%, respectively). BSU accreditation ensures the presence of training in all modalities of treatment of urinary lithiasis. In relation to the number of programs that offered fellowships in endourology (Internship and Improvement Programs) only 3 were accredited by BSU, 1 in South region (Santa Catarina State) and 2 in Southeast (in São Paulo State). Finally, in relation to the number of urologists/inhabitants, Southeast region presented the higher proportion (1: 31, 745 inhabitants), and the North and Northeast region had the lowest number of professionals (1: 90.095 and 1: 70.771 inhabitants, respectively). All data are presented in detail in Table-1.

**Table 1:** Urology Demographic Data in Brazil.

	South	Southeast	Center-west	Northeast	North	Total
Total number of urologists	900 (16.9%)	2.781 (52.2%)	533 (10%)	879 (16.5%)	229 (4.3%)	5.328
Urologists members OF BSU	737 (15.5%)	2.739 (57.5%)	279 (5.9%)	809 (17.3%)	181 (3.8%)	4.745
BSU urologists/Total	81.9%	98.5%	52.3%	92%	79%	89.1%
Total number OF MRU	15 (12.3%)	70 (57.4%)	10 (8.2%)	22 (18%)	5 (4.1%)	122
BSU Accredited MRU	12 (15%)	47 (58.7%)	7 (8.8%)	13 (16.2%)	1 (1.3%)	80
BSU MRU/Total	80%	67.1%	70%	59.1%	20%	65.6%
Urologists/Inhabitants	1: 40.223	1: 31.745	1: 56.903	1: 70.771	1: 99.095	

**MRU** = Medical Residence in Urology; **BSU** = Brazilian Society of Urology

### Current status of endourology in Brazil

A total of 1.267 urologists answered the questionnaire, corresponding to 26.7% of all members of BSU (5.328). Among the respondents, 53.3% lived in the Southeast region (674), 17.9% in South region (227), 15.4% in Northeast (195), 8.5% in Center-West (107) and 4.9% in the North region (62). The percentage of BSU members per region that answered questionnaire was 24.6% in the Southeast, 26.4% in the North, 30.8% in the South, 38.3% in the Center-West and 34% in the Northeast.

In general, most urologists perform the three most important endourological procedures for the treatment of kidney stones, especially URS (95.6%), while 80.2% perform F-URS and 72.1% PCNL. In relation to number of procedures, the majority of urologist perform 0-10 endourological procedures/month (73.4% to 88.2%), and F-URS was the least performed (11.7% of >10 procedures/month). In relation to the reasons for a low number of procedures or for not performing the technique, most informed that there was a lack of patients with the disease (42.1% to 67.7%). When lack of patients was excluded as a determinant factor, lack of equipment or adequate hospital infrastructure was the main reason for URS (23%) and F-URS (38.1%), while for PCNL the lack of practical experience was the main reason (29.9%).

Finally, most urologists expressed interest in participating in hands-on endourological courses in their region (72%) and most would travel more than 50 km to attend them (73.5%). Likewise, many respondents (42.9%) would travel more than 200 km to participate in these events.

### Regional characteristics of endourology in Brazil

The answers of this research were analyzed and compared among different regions in Brazil and data are presented in Table-2.

**Table 2:** Answers of Respondents.

		Southeast	Northeast	South	CenteR-west	North	P
**Do you perform rigid ureterolithotripsy?**							**0.953**
Yes		95.7%	95.9%	95.6%	95.3%	95.3%	
**How often do you perform r-ult?**		**a**	**b**	**b**	**b**	**a, b**	**0.001**
0 to 10		68.5%	78.9%	77.8%	79.6%	82.8%	
10 to 20		23.2%	18.3%	19.9%	18.4%	15.5%	
>20		8.3%	2.8%	2.3%	1.9%	1.7%	

**Reason for low use of rigid ult**							**0.069**
	1 Lack of equipment/ infrastructure of my hospital	17.6%	31.5%	26.3%	13.6%	40%	
	2 Lack of patients with the disease	73%	56.2%	68.4%	77.3%	48.6%	
	3 Lack of practical experience with the technique	7.7%	7.9%	4.2%	9.1%	8.6%	
	4 Lack of theoretical knowledge of the technique	0.5%	0%	0%	0%	0%	
	5 Lack of trained support staff (assistants, nurses, etc.)	1.4%	4.5%	1.1%	0%	2.9%	

**Do you perform flex-ult?**		**a**	**b**	b	**b**	**a, b**	**<0.001**
	Yes	85.9%	73.8%	72.7%	71%	80.6%	

**How often do you perform flex-ult?**		**a**	**a**	**a**	**b**	**a, b**	**0.028**
	0 a 10	86.2%	88.9%	92.6%	89.7%	92%	
	10 a 20	11.4%	9.7%	6.9%	3.8%	8%	
	>20	2.4%	1.4%	0.6%	6.4%	0%	

**Reason for low number of flex-ult?**		**a**	**a, b**	**b**	**a, b**	**a, b**	**0.011**
	1 Lack of equipment/ infrastructure of my hospital	29.3%	42%	51.2%	44.3%	47.5%	
	2 Lack of patients with the disease	52.8%	37.5%	33.3%	41%	37.5%	
	3 Lack of practical experience with the technique	15.9%	17%	14%	14.8%	12.5%	
	4 Lack of theoretical knowledge of the technique	1.4%	2.7%	0%	0%	0%	
	5 Lack of trained support staff (assistants, nurses, etc.)	0.7%	0.9%	1.6%	0%	2.5%	

**Do you perform PNL?**		**a**	**a**	**b**	**c**	**a**	**<0.001**
	Yes	73.7%	69.1%	82.4%	48.6%	67.7%	

**How often do you perform PNL?**							**0.642**
	0 to 10	77%	80.3%	80.7%	79.6%	79.5%	
	10 to 20	21%	19.7%	16.6%	18.5%	20.5%	
	>20	2%	0% ^a^	2.7%	1.9%	0%	

**Reason for low number of PNL?**		**a**	**b**	**c**	**b**	**b**	**<0.001**
	1 Lack of equipment/ infrastructure of my hospital	16.2%	32%	25.6%	26.6%	40%	
	2 Lack of patients with the disease	47.7%	29.7%	57%	31.6%	24.4%	
	3 Lack of practical experience with the technique	33.2%	32%	13.2%	34.2%	33.3%	
	4 Lack of theoretical knowledge of the technique	1.1%	3.1%	0.8%	6.3%	0%	
	5 Lack of trained support staff (assistants, nurses, etc.)	1.7%	3.1%	3.3%	1.3%	2.2%	

**Would you participare in courses in your region?**							**0.860**
	Yes	72.5%	73.3%	72.2%	67.3%	71%	

**Which distance would you travel?**		**a**	**b**	**c**	**b**	**d**	**<0.001**
	1 - 0-50 Km	33.1%	17.4%	22.7%	19%	14.5%	
	2 - 50 - 200 Km	35.6%	23.7%	34.1%	21%	4.8%	
	3 - > 200 Km	31.3%	58.9%	43.3%	60%	80.6%	

**Rigid ULT** = semi-rigid ureterolothotripsy; **Flex-ULT** = flexible ureterolithotripsy; **PNL** = percutaneous nephrolithotomy

* Equal letters inside the same line represent absence of significant statistical difference.

When we analyze the practice of URS by region, no statistical difference was found in the percentage of urologists that perform the technique (95.3-95.7%, p 0.953); however, when we analyzed the frequency of procedures according to region, there was a higher proportion of urologists from Southeast that performed >20 procedures/month (8.3% vs. 1.7-2.8% p 0.001). In all regions, the main reason for not performing URS was the lack of patients (48.6%-77.3%), however, there was a higher percentage of lack of equipment/hospital infrastructure in North and Northeastern regions when compared to the others, although not significantly different (40% and 31.5% respectively vs. 13.6-26.3%, p=0.069).

In relation to F-URS, the percentage of urologists that performed the technique was significantly higher in the Southeast and North regions that in Northeast, South and Center-West (85.9% and 80.6% vs. 73.8%, 72.7% e 71% respectively, p <0.001). In all regions, there was a predominance of <10 procedures/month (86.2%-92%), while in Center-West there was a significantly higher percentage of urologists performing >20 procedures/ month (6.4% vs. 0-2.4%, p 0.028). In relation to the main reason for a lack or low number of procedures, lack of patients predominated only in the Southeast region (52.8% vs. 33.3-41%), while in all other regions the lack of equipment/hospital infrastructure (42-51.2%) was the most important factor, although there was only a statistical significant difference between the Southeast and South regions (p=0.011).

In relation to PCNL according to Brazilian region, there was a statistical significant difference between the rate of urologists that claimed to frequently perform this procedure, with a prevalence in the South region (higher number) and lowest frequency in the Center-West region (82.4% and 48.6% respectively vs 67.7-73.7%, p <0.001). In relation to the number of procedures, there was no significant difference among the different regions, most stating that performed <10 procedures/ month (77% to 80.7%, p 0.642). The main reason for this low number of PCNLs was lack of patients particularly in South and Southeast regions (57% and 47.7%), lack of equipment/hospital infrastructure mainly in North and Northeast region (40% and 32%) and the lack of practical experience in the Center-West region (34.2%). Surprisingly, except in the South region, in all other regions there were many urologists pointing the lack of experience with the technique as one of the main reasons for not performing PCNL (13.2% vs. 3234.2%, p <0.001). In Center-West and Northeast regions, there was also a higher proportion of urologists that referred lack of theoretical knowledge of the procedure (6.3% and 3.1% respectively vs. 0-1.1%, p <0.001).

Finally, when the respondents were questioned about practical courses of endourology, in all regions there was a higher proportion of interested urologists (67.3-72.5%, p 0.86). In relation to the distance that they would travel to attend them, there was a statistical significant different (p <0.001) between those who would travel large distances (>200 km), as the Southeast region presented the lowest rate (31.3%), followed by the South Region (43.3%), while the urologists from the North region were those with higher rate of interest (80.6%).

## DISCUSSION

In Brazil and Worldwide, the prevalence of nephrolithiasis has increased over the years in the last decades, with a major impact on public health systems (1-5). This increase has been accompanied by the growing use of surgical treatments for kidney stones in developed and underdeveloped countries (9, 12, 15, 16), increasing the importance of a better understanding of the current practices of endourology. Our study has investigated the three most important surgical techniques used for the treatment of nephrolithiasis among Brazilian urologists. To our knowledge, this is the first study to specifically address the demography of endourology in Brazil.

After reviewing the answers of the question forms, it was possible to conclude that URS, rigid or flexible, is well established in Brazil, since more than 80% of the respondents claimed to perform this procedure in their daily practice, regardless of their region. However, PCNL is still less performed by Brazilian urologists (72%) in comparison to ureteroscopy, probably due to its higher complexity and the risk of more severe complications, such as sepsis, injury of adjacent organs, hemorrhage and death, therefore generally being reserved for more complex cases. The fact that lack of patients corresponds to the main reason why most urologists perform <10 surgeries/month suggests that, in Brazil, endourology is decentralized (too many surgeons operating few patients), and that there is a lack of reference centers for the treatment of nephrolithiasis.

Lack of equipment or hospital infrastructure apparently are the main obstacles for the practice of Ureteroscopy in all of Brazilian regions. In relation to URS, that that most urologists perform routinely (95% in all regions), the lack of material/infrastructure is a major hindrance, particularly in North and Northeast regions (40% and 31.5%, respectively), precisely those with the lowest HDI (14) and the least amount of health resources. Regarding F-URS practice, in all regions more than 70% of urologists perform the procedure (the technique is well established in Brazil), but the lack of equipment is still a factor to prevent higher number of surgeries (29.3%-51.2%), probably due to the high costs necessary for the equipment acquisition and maintenance, as well as for the short lifespan of flexible ureteroscope and the need of laser devices to treat stones. Lack of resources was less impactful in Southeast than in other regions (29.3% vs. 42-51.2%), probably due to higher HDI rates, higher number of urologists, more RMU programs and endourology fellowships, so more resources are available to provide the use of edge technologies in health care. Recent actions of BSU, such as the recent incorporation of URS in public health services, may contribute to change this reality in Brazil, as it may lead to the destination of more equipment and hospital infrastructure improvements along the country, reinforcing the role of urologists in national health care politics.

This study also highlights the deficiency that still exists in Brazil regarding that practice of PCNL. In most Brazilian regions, except South, only 48-73% of respondents claimed to perform PCNL, being lack of practical experience the main reason (32-34%). These data are in accordance with another Brazilian study that also concluded that the lack of training was the main reason for the low use of PLN (17). In that study, most urologists that performed the technique were young, with adequate training during their medical residency, reinforcing the importance of MRU programs to prepare urologists to perform PCLN. As in ureteroscopy, lack of equipment/hospital infrastructure were observed in the regions with lower HDI (40% North and 32% Northeast), probably due to lower investments in health and education. On the other hand, in South, PCNL is performed by 82% of respondents and only a small proportion of those (13.2%) claimed lack of practical experience with the technique, probably due to a higher number of MRU and urological services that teach and perform PCNL. These results emphasize the need to encourage PCNL teaching programs by hands-on courses or by increasing of the number of procedures during residency. Creating of more referral centers and endourology fellowship programs, particularly in regions other than the South and Southeast, may improve this scenario, since with more procedures the rate of complications and clinical results greatly improves (18, 19).

Finally, this study has shown that most Brazilian urologists are interested in participating in endourology courses (>70% in all regions), and, except in South and Southeast, most urologists would travel more than 200 km to attend these courses, probably due to the fact that most are located in those more developed regions, with better infrastructure and higher concentration of urologists.

However, the results of our study must be interpreted carefully, and some limitations must be taken into account: it was based on a volunteer electronic question form, only 25% of BSU members answered the questionnaire, there may have been a higher participation of younger urologists, those from academic centers or those with more expertise in endourology, that could have overestimated the percentage of urologists familiar with those techniques to surgically treat kidney stones, with a higher number of procedures. On the other hand, due to the same reasons, since many respondents indicated lack of practical experience in PCNL, this information is even more relevant, since there could be many more general urologists that need training. Also, it was not possible to determine if the respondents practiced mainly in private or public health services, what could have influenced surgical daily practice. Usually, public services suffer from lack of resources and surgical material, while private services usually perform more procedures with lower morbidity potential and higher remuneration, such as flexible ureteroscopy instead of PCNL. Other aspects were not addressed, such as age influence and remuneration of the procedures, as well as availability of extracorporeal lithotripsy as an alternative method to treat kidney stones. All these aspects should be evaluated in future studies but, nevertheless, the results of this study reflect the status of a great proportion of Brazilian urologists equally distributed in the country, providing important information about the current scenario of endourology in Brazil. More studies sponsored by BSU should be encouraged to deepen the understanding of endourology practices in our country, providing data to propose new courses and actions directed to improve the main deficits of this area.

## CONCLUSIONS

This study was able to present important information regarding current scenario of endourology in Brazil. Ureteroscopy, flexible or rigid, are well stablished in the country, and more resources are necessary to improve their practice, mainly in underdeveloped regions. Regarding PNL, a significant part of Brazilian urologists still have no practical experience with the method, and more teaching efforts should be made.

## ABBREVIATIONS

PCNL: percutaneous nephrolithotomy
URS: rigid ureteroscopy
F-URS: flexible ureteroscopy
MRU: Medical Residency in Urology
BSU: Brazilian Urological Society
